# Middle ear ventilation status postoperatively after translabyrinthine resection of vestibular schwannoma with mastoid obliteration and Eustachian tube occlusion: is the Eustachian tube enough to ventilate the middle ear without the mastoid air cell system?

**DOI:** 10.1186/s40463-016-0157-z

**Published:** 2016-08-30

**Authors:** James Belyea, Brandon Wickens, Manohar Bance

**Affiliations:** Division of Otolaryngology-Head & Neck Surgery, Department of Surgery, Dalhousie University, Room 3184 Dickson Building, VGH Site, 1278 Tower Road, QEII HSC, Halifax, NS B3H 2Y9 Canada

**Keywords:** Eustachian tube, Tympanogram, Mastoid air cell system, Middle ear ventilation, Pressure regulation

## Abstract

**Background:**

Gas pressure balance is essential for maintaining normal middle ear function. The mucosal surfaces of the middle ear, the mastoid air cell system (MACS), and the Eustachian tube (ET) play a critical role in this process; however, the extent that each of these factors contributes to overall middle ear ventilation is unknown. The objective of this study was to determine if the ET alone can maintain normal middle ear pressure without the MACS. To do this, we reviewed subjects who had their MACS completely removed with translabyrinthine (TL) surgery for vestibular schwannoma.

**Methods:**

A retrospective chart review was done to collect pre and postoperative tympanometry data from patients who underwent resection of vestibular schwannoma. Data from the operative side was compared to the non-operative side at 2 years post-op.

**Results:**

Twenty-four patients were included in this study. Of these, 63 % achieved a type A tympanogram at 2 years post-op in the TL resection group, implying an ability to maintain middle ear pressure in the absence of a mastoid cavity. Because some had negative pressures post TL resection, the average change in pre and postoperative pressure was -37.5 daPa for the operative side and 7.8 daPa for the non-operative side. This was significantly different.

**Discussion:**

The difference for change in pre and postoperative pressure and compliance between operative and non-operative side might be expected from the ET plugging during TL resection. However, more interesting are those patients in whom the ET presumably reopens, and in these subjects, despite having no mastoid compartment at all, and the space obliterated with fat, they were still able to maintain normal ventilation of the middle ear space.

**Conclusion:**

Our findings imply that the ET alone is adequate to ventilate at least the reduced middle ear space following TL surgery in most subjects, and perhaps in 100 % if the ET hadn’t been plugged during surgery. Hence, the mastoid air cell system, even when healthy, is not needed to maintain air in the middle year cleft.

## Background

It is well recognized that middle ear ventilation is essential for the middle ear to function as an impedance matching transformer, and so is needed to achieve good hearing results [1]. Gas pressure balance is required to maintain middle ear (ME) function and the mucosal surfaces of the ME, the mastoid air cell system (MACS) and the Eustachian tube (ET) all play an important role in this process [2]. While the ET is usually considered the main mechanism for equalizing extraordinary pressure changes, for instance diving or flying, the MACS is relevant for day to day pressure equalization at relatively normal ambient pressures [3]. The mastoid mucosal surface participates in this though a process of middle ear gas exchange [4]. Gaihede et al. showed that the human MACS, as well as the ET were capable of active counter-regulation of the middle ear pressure in short term pressure changes in healthy ears and were able to function in a complementary way [5]. The MACS may also have a role as a pressure buffer function [3, 6]. Overall, the role of the MACS is complex, and in many respects poorly understood. It may act as a temperature buffer from the environment to shield the labyrinth [3], in addition to helping to regulate the pressure changes in the middle ear. Magnuson has pointed out that gas exchange across the MACS results in a slow negative pressure development during the awake cycle, interrupted by increases in pressure as the ET opens [3, 7–9]. During sleep, however, the cycle is reversed, as the MACS produces a slow increase in pressure, as result of increasing CO2 and transudation across the capillary structure, which is punctuated by drops in pressure as the ET opens [3, 7–9]. Indeed, the mastoid portion of the MACS seems very different than the tympanic portion of the air cell system, and specialized in many ways for gas exchange. The pro/meso/hypotympanum maybe specialized for mucociliary clearance, and is lined with pseudostratified and ciliated epithelium, with abundant mucous producing cells [4]. In contrast, the mastoid part is lined with a highly vascularized monocellular layer [4]. Recently, it has been reported that the vascular supply to the mastoid mucosa is very specialized, with numerous micro-channels that may connect to the mastoid surface [10].

Lack of the pressure buffer function of the mastoid also leaves the middle ear vulnerable to the effects of environmental pressure changes [2]. In fact Csakanyi et al. based on model calculations, have suggested that when the mastoid size is between 3 and 6 ml, removing the mastoid by obliteration actually tips the balance between gas exchange and large volume pressure buffer towards a more stable pressure [2]. Larger mastoids are protected by their volume acting as a pressure buffer [2].

With this degree of complexity, it is unclear what the relative contributions are of the ET and MACS to overall ME ventilation, and if the ET alone can adequately ventilate the ME after obliteration of the MACS in the long term. This is becoming increasingly important to answer with the recent popularization of mastoid obliteration surgeries for cholesteatoma [11–14], first introduced by Mercke [15]. In these techniques, the mastoid is completely obliterated with bone pate, to prevent recurrence of cholesteatoma in this region. However, this leaves the residual middle ear completely dependent on ventilation through the ET, and ventilation is vital to achieve any degree of success for hearing reconstruction.

Various studies have attempted to examine the relative contributions of the mastoid mucosal system and the ET in middle ear ventilation to explain this, but none have provided a definitive answer [3, 5, 16].

Our basic question was could a middle ear system with no MACS maintain adequate middle ear ventilation in the longer term, purely with the ET alone? We felt that patients who had translabyrinthine (TL) resection of vestibular schwannoma provided us with a model to test this question. This model is particularly suited, since as opposed to chronic ear disease after mastoid obliteration, almost all cell tracts are completely removed and then obliterated with fat. Also, the contralateral ear is healthy, so can serve as control for normal physiology, as opposed to chronic ears with obliteration, in which there is often bilateral disease. As a routine step in TL resection of vestibular schwannoma, the ET is occluded with a muscle plug, to minimize potential post-op cerebrospinal fluid leak. Over time, the muscle plug is likely displaced, and the ET opens up in some patients, but the mastoid system never reconstitutes itself. ME pressure thereby has to be maintained with the ET alone. We would expect that in some patients, the ET remains blocked, and would not function, but if any patients could develop normal middle ear pressures after removal of the MACS, it would be of interest.

In order to further assess the role of the ET in middle ear ventilation, a retrospective chart review was conducted on patients who underwent TL resection of vestibular schwannoma with MACS obliteration and ET occlusion. Pre and postoperative tympanometry data was used to compare the operative and non-operative (control) side.

## Methods

### Study design

Retrospective chart review.

### Patients

A comprehensive review of all patients assessed through the Maritime Lateral Skull base clinic at the Queen Elizabeth II Health Sciences Center, Halifax, Nova Scotia from 2001 to 2013 was conducted. Inclusion criteria included all adult patients who underwent TL resection of vestibular schwannoma with MACS obliteration and ET occlusion. Exclusion criteria were skull base or Cerebellopontine angle tumors other than vestibular schwannoma, approaches other than the TL approach, pre/postoperative radiotherapy, abnormal preoperative tympanometry on either side, revision surgery or previous mastoidectomy, and incomplete data. Data collected included gender, age at time of procedure, pre and postoperative tympanometry data for both the operative and non-operative side. The non-operative side served as a control group. Preoperative pressure, compliance and tympanogram type were compared with tympanometry data from 2 years postoperatively. The average change in pre/postoperative pressure and compliance was calculated for the operative and non-operative side. A paired Student’s T-Test was used to compare the average change in pre/postoperative pressure and compliance of the operative and non-operative sides. A *p*-value less than 0.05 was considered significant. Pre and postoperative tympanograms were noted for both the operative and non-operative sides. The time interval post-op for tympanogram normalization was also explored.

## Results

### Demographics

Thirty-five patients met inclusion criteria. Eleven had to be excluded due to incomplete data, leaving 24 patients included in this study. There were 10 male and 14 female patients. Average age was 52 years with a range of 17–73 years. Four of the 24 patients experienced a postoperative CSF leak.

### Tympanometry data—pressure

**Fig. 1:**
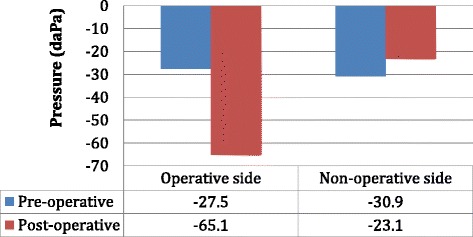
Tympanometry data showing the average pre and postoperative pressure of the operative and non-operative sides

**Fig. 2:**
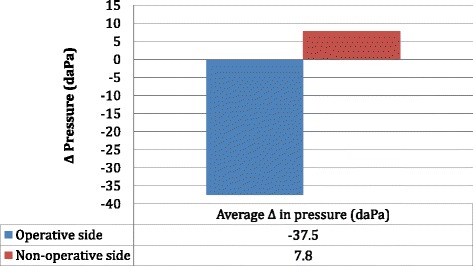
Tympanometry data showing the average change in pre and postoperative pressure of the operative and non-operative sides. Statistically significant difference between groups (*p* = 0.00001)

The average preoperative pressure of the operative and non-operative sides was -27.5 daPa and -30.9 daPa, respectively (Fig. 1). The average postoperative pressure of the operative and non-operative sides was -65.1 daPa and -23.1 daPa, respectively (Fig. 1). The average change in pressure of operative and non-operative sides was -37.5 daPa and 7.8 daPa, respectively and there was a statistically significant difference between groups (*p* = 0.005) (Fig. 2).

### Tympanometry data—compliance

**Fig. 3:**
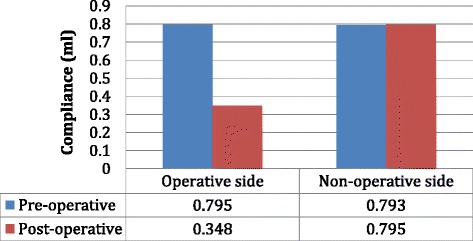
Tympanometry data showing the average pre and postoperative compliance of the operative and non-operative sides

**Fig. 4:**
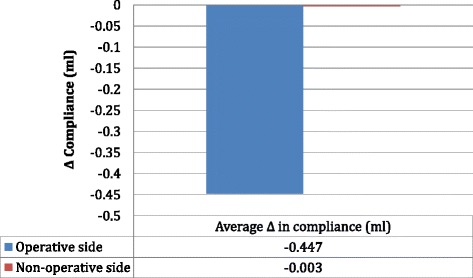
Tympanometry data showing the average change in pre and postoperative compliance of the operative and non-operative sides. Statistically significant difference between groups (*p* = 0.014)

The average preoperative compliance of the operative and non-operative sides was 0.795 ml and 0.793 ml respectively (Fig. 3). The average postoperative compliance of the operative and non-operative sides was 0.348 ml and 0.795 ml, respectively (Fig. 3). The average change in compliance of operative and non-operative sides was -0.447 ml and -0.003 ml respectively and there was a statistically significant difference between groups (*p* = 0.00009) (Fig. 4).

### Tympanometry data—tympanograms

**Fig. 5:**
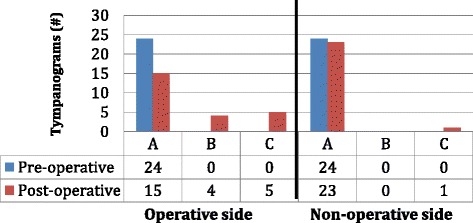
Tympanometry data showing number and types of tympanograms pre and post-op for the operative and non-operative sides

All 24 patients had normal type A tympanograms on both the operative and non-operative sides pre-op (Fig. 5). On the non-operative side, 23/24 (96 %) patients had a type A tympanogram 2 years postoperatively. On the operative side, 15/24 (63 %) patients had a type A tympanogram 2 years postoperatively (Fig. 5), and these are the patients of interest to us. Of the 15 patients who had a type A tympanogram on the operative side 2 years postop, 4/15 (27 %) normalized by 6 months postop, a further 6/15 (40 %) normalized between 6 months and 1 year postop, a final 5/15 (33 %) normalized between 1 and 2 years post op (Table 1). Of the 15 patients who had a type A tympanogram on the operative side 2 years postop, the average pre-op middle ear pressure was -25.7 daPa, the average post-op middle ear pressure was -26.6 daPa and the average change in middle ear pressure was -0.9 daPa.

**Table 1:** Timing of normalization of post-op tympanograms

Time interval post-op	Number of patients	Percent of patients
Less Than 6 Months	4/15	27 %
6–12 Months	6/15	40 %
12–24 Months	5/15	33 %

## Discussion

Ventilation of the ME is a complex process involving the ET, MACS, and mucosal surface of the ME; however, the amount that each of these contributes to overall ME ventilation is unknown. The main striking finding in our study is that 63 % of our patients with a completely missing MACS system were able to maintain a type A tympanogram at 2 years post-surgery, with the ET alone. It is possible that if we had picked a long follow-up period, more would have normalized, but 2 years seemed a reasonable compromise with “long term” data and getting sufficient numbers to test. These results clearly show, that at least in some ears, the middle ear is able to maintain ventilation of at least the reduced middle ear cleft after MACS removal, even after 2 years. Of the remaining 37 % who did not achieve a type A tympanogram, our suspicion is that in these patients the ET plugging from surgery had not become patent. An alternative explanation could be that the ET was open, but that some ears require both a MACS and a ET for best middle ear function. While most studies show that the primary source for gas exchange is in the mastoid mucosa, which seems specialized for this function, it is possible that some gas exchange occurs through the hypotympanic air cells [10]. We cannot absolutely rule this out, but consider it unlikely.

Our study showed a statistically significant difference for change in pre and postoperative pressure and compliance between the operative and non-operative sides. This is hardly surprising, as the operative side had ET plugging during surgery. This is in keeping with a similar study by Chiossone-Kerdel et al from 2002, who reviewed 42 patients with TL resection of vestibular schwannoma with ET occlusion and MACS obliteration and found a significant difference in tympanogram volumes and pressures within 1 year postoperatively between the operative and non-operative side, which continued for volume, but not pressure at more than 1 year post-op [17].

Our study does not investigate how the middle ear copes with pressure stresses, such as flying or diving, without a MACS. The MACS acts as a pressure buffer, and presumably pressure changes in the middle ear would be larger for a given change in ambient pressure then they would be for a fully developed MACS system [2, 3]. However, direct questioning of our patients does not reveal any subjective complaints about pain on flying etc. Also, without the MACS, there may be a difference in the diurnal and daily changes in the pressure in the middle ear. On the one hand, the lack of a MACS means there is less diffusion of CO2 into the middle ear, as this gas rises in the bloodstream during sleep and from transudation through the MACS capillary system with recumbancy [3]. On the other hand, there is less pressure buffering without the mastoid volume, sniffing or other evacuations [3]. Alternatively, influxes of air through the ET cause larger pressure changes. Patients with TL resection may not notice the effects of any such changes on their hearing as they are deaf, but it is possible that subjects with mastoid obliteration for chronic ear disease might. Future studies should focus on the ability of the ET alone to maintain a stable pressure during the day and night, without a MACS; particularly, the differences in pressure variations during the daily cycle between the TL resection side and the normal side.

While our study shows that at least in some ears, the Eustachian tube alone is sufficient to ventilate the middle ear, it does not address if these reduced cavity middle ears have more fluctuation in middle ear pressure than normal ears, as they have lost the volume of the mastoid as a pressure buffer. This would require longitudinal studies of middle ear pressure over several hours and days.

## Conclusion

Although there was a statistically significant difference for change in pre and postoperative pressure and compliance between the operative and non-operative sides, a large portion of postoperative patients achieved a normal Type A tympanogram. In our group of patients, the ET alone was able to ventilate the middle ear space and the role of the MACS in maintaining ME pressure was not clear.

## Abbreviations

ET: Eustachian tube
MACS: Mastoid air cell system
ME: middle ear
TL: Translabyrinthine
